# Psychological readiness for return to sport following distal femoral osteotomy in patients with recurrent patellar instability

**DOI:** 10.1186/s12891-025-08348-x

**Published:** 2025-03-31

**Authors:** Maximilian Hinz, Peter Rab, Moritz Brunner, Marco-Christopher Rupp, Armin Runer, Luca Grüning, Lisa Rahn, Sebastian Siebenlist, Andrea Achtnich

**Affiliations:** 1 Department of Sports Orthopaedics, Technical University of Munich, Ismaninger Strasse 22, 81675 Munich, Deutschland, Germany; 2 Department of Trauma Surgery, Regensburg University Medical Center, Regensburg, Germany

**Keywords:** ACL-RSI, Return to sport after injury, Derotational osteotomy, Varus osteotomy, Maltracking

## Abstract

**Background:**

There exists a paucity of data on the relationship between psychological factors and return to sport in patients who undergo surgery for complex patellofemoral instability (PFI). The purpose of this study was to investigate the influence of psychological factors on the return to the preoperative level of sports and knee function in patients with complex PFI who were treated with distal femoral osteotomy (DFO).

**Methods:**

Patients who underwent DFO for recurrent PFI associated with increased femoral antetorsion and/or valgus malalignment were included. Psychological readiness to resume sporting activities was assessed at a minimum of 12 months postoperatively using the PFI-Return to Sport after Injury (PFI-RSI) scale. A receiver operating curve (ROC) analysis was performed for the PFI-RSI scale and its ability to discriminate between patients who returned to the preoperative level of sport and those who did not. Spearman’s rank-order correlation was used to test for correlations between the PFI-RSI scale and patient-reported outcome measures (PROM), including Banff Patella Instability Instrument 2.0 (BPII 2.0), Kujala score, Tegner Activity Scale (TAS) and Visual Analog Scale (VAS) for pain.

**Results:**

Sixty-five patients (70.8% female) were included at a median of 61.0 months (40.0-78.5 months) postoperatively. Patients who returned to their preoperative level of sports scored significantly higher on the PFI-RSI scale than patients who did not (75.8 [64.4–84.2] vs. 40.8 [23.4–60.9], *p* < 0.001). Reaching a threshold value of 55 on the PFI-RSI scale could predict whether or not patients returned to the preoperative level of sport with a sensitivity of 90.9% and a specificity of 70.6% (area under the curve = 0.834; Youden index = 0.615). The PFI-RSI scale showed moderate to strong correlations with PROM.

**Conclusion:**

Psychological readiness to resume sporting activities correlated with knee function and was significantly higher in patients who achieved the preoperative level of sport than in patients who did not.

## Background

Patients with patellofemoral instability (PFI) often suffer from a poor activity level and quality of life due to physical limitations and loss of confidence [1]. Also, PFI may lead to increased activity in brain areas associated with anxiety or fear [2]. Although reconstruction of the medial patellofemoral ligament (MPFL) is generally associated with a good functional outcome and high return to sport rates, existing anxiety and depression levels may not improve [3, 4].

Further, psychological factors have been identified as the main cause in patients for not resuming sporting activities following MPFL reconstruction [5, 6]. The MPFL-Return to Sport after Injury (MPFL-RSI) scale is a modification of the Anterior Cruciate Ligament-Return to Sport after Injury (ACL-RSI) scale that assesses emotions, confidence in performance and risk appraisal [6–8]. Hurley et al. [6] reported that the majority of patients who did not return to sport following MPFL reconstruction presented with poor psychological readiness to resume sporting activities and that fear of re-injury was the most common reason for patients to not return to sports.

There exists, however, a paucity of data on the psychological readiness for the return to sports that patients with high-grade PFI have after undergoing more complex procedures, such as osteotomies, that may be necessary to improve patellofemoral alignment [9, 10]. Therefore, the purpose of this study was to report on the sporting ability and the reasons as to why athletes do not return to their preoperative level of play following distal femoral osteotomy (DFO) for the treatment of high-grade PFI. It was hypothesized that (1) patients who returned to their preoperative level of sport would show a significantly higher psychological readiness than patients who did not, (2) the PFI-RSI scale could discriminate between patients who did and those who did not return to the preoperative level of sport and that (3) psychological readiness would correlate with functional outcomes.

## Methods

The present study was approved by the ethics committee of the Technical University of Munich (reference: 2022-193-S-NP) and conducted according to the Declaration of Helsinki. Patients who underwent a varus-producing and/or derotational DFO at our institution for the treatment of recurrent PFI (≥ 2 patellar dislocations) and valgus malalignment and/or increased femoral antetorsion were eligible to participate (minimum follow-up: 12 months). Only patients with a minimum age of 16 years at follow-up were eligible to participate.

### Radiological parameter measurement and surgical planning

For the preoperative analysis of coronal limb alignment, the femorotibial angle (FTA), mechanical lateral distal femoral angle (mLDFA) and mechanical medial proximal tibial angle (mMPTA) were analyzed on weight-bearing whole-leg anteroposterior radiographs using the medical software mediCAD^®^ (accuracy: 0.01°; mediCAD Hectec GmbH, Altdorf, Germany) according to the method proposed by Strecker [11].

Lower extremity torsion was measured according to a method previously described by Schneider et al. [12]. Femoral antetorsion was defined as the angle between a line connecting the center of the femoral head and the center of the distal femoral neck (femoral neck axis) and the distal femur (dorsal margin of the femoral condyles). External tibial torsion was defined as the angle between a tangent drawn along the dorsal margin of the proximal tibia and, distally, a line connecting the center of the tibial pilon with the center of a line across the fibular incision of the distal tibia.

A DFO was indicated in patients suffering from recurrent PFI with closed physes and associated valgus > 3° and/or femoral antetorsion > 20°. Additional underlying structural risk factors were addressed via additional procedures, including MPFL reconstruction, trochleoplasty and tibial tuberosity transfer based on the individual risk factor analysis.

### Surgical technique

Diagnostic arthroscopy was performed first in all patients to evaluate the cartilage, patellar tracking and the integrity of the medial retinaculum. In cases of a high lateralization tendency following DFO, an additional reconstruction of the MPFL using the ipsilateral gracilis tendon was performed [13].

A biplanar supracondylar osteotomy of the femur was performed via a standardized lateral subvastus approach. A detailed description of the authors’ preferred operative techniques were previously described both for the varus-producing [14] and derotational techniques [15]. As previously reported, a combined varization and derotation may also be performed using the same biplanar supracondylar osteotomy [16].

The aim was to achieve neutral alignment for varus-producing DFO and physiological femoral antetorsion for derotational DFO. An internal plate fixator system with locking screws was used (TomoFix^®^ Lateral/Medial Distal Femur Plate, DePuy Synthes, Raynham, Massachusetts, USA) for the fixation of the osteotomy.

### Postoperative rehabilitation

Postoperatively, partial weight bearing (20 kg) for six weeks was allowed. The degree of range of motion restriction was dependent on the additional surgical procedures. If an additional MPFL reconstruction was performed, range of motion was limited to 90° of flexion for the first six weeks and a knee brace was worn (M.4s^®^, medi Bayreuth, Germany). After a check-up 6 weeks postoperatively, full weight bearing was encouraged. Physical therapy started on the first postoperative day with passive flexion with patients receiving treatments 2–3 times per week.

### Patient characteristics and operative data

Chart review was performed to obtain patient characteristics (e.g., age at the time of surgery, sex) and operative data (e.g., type of osteotomy, concomitant procedures, intra- and perioperative complications).

### Outcome measurements

Patient-reported outcome measures (PROM), including the PFI-RSI scale [6], Banff Patella Instability Instrument 2.0 (BPII 2.0) [17], Kujala score [18], Tegner Activity Scale (TAS) and Visual Analog Scale (VAS) for pain as well as subjective satisfaction with the postoperative result (1–10 scale with “10” indicating maximum satisfaction) were obtained. The PFI-RSI scale assessed psychological readiness to return to sporting activities on a scale of 0 to 100 with higher values indicating better psychological readiness. The PFI-RSI scale is a modification of the validated ACL-RSI scale which had previously been adapted for various other pathologies, including patients undergoing MPFL reconstruction [6, 19–22]. The BPII 2.0 is a 23-item questionnaire that evaluates the quality of life in patients with PFI on a scale of 0 to 100 with higher values indicating better quality of life [17]. The Kujala score is a 13-item questionnaire that assesses symptoms and functional limitation in patients with patellofemoral disorders on a scale of 0 to 100 with higher values representing fewer symptoms and better knee function [18]. The TAS evaluates the activity level on a scale of 0 to 10 with higher values indicating higher activity level [23]. The VAS for pain assesses pain intensity on a scale of 0 to 10 with “0” indicating no pain and “10” indicating worst pain [24].

Furthermore, data on the return to sporting activities were collected. In patients that did not reach the preoperative level of sports, reasons for a postoperative decrease in sporting ability reasons were evaluated, which encompassed: (1) fear of re-injury, (2) knee pain, (3) feeling of instability, (4) lack of confidence in the knee joint, (5) other injury-dependent factors and (6) other reasons.

Lastly, rates of the patellar re-dislocation and revision surgery were recorded.

### Statistical analysis

Data were analyzed using SPSS 26.0 (IBM-SPSS, New York, USA). Data for one knee per patient were reported and considered for statistical analyses. Categorical variables are presented in number and percentages. Normal distribution of the collected continuous variables was assessed using the Kolmogorov-Smirnov test and graphically confirmed. Normally distributed continuous variables are reported as mean ± standard deviation whereas non-normally distributed continuous variables are shown as median (25–75% interquartile range). For group comparisons of continuous variables, the Wilcoxon test or *t* test was applied and for group comparisons of ordinal variables, the Mann-Whitney U test was applied.

The receiver operating characteristic (ROC) curve method [25] was used to assess the classification accuracy of the PFI-RSI scale on whether or not return to preoperative level of sport was possible. Subsequently, the area under the curve was assessed. The Youden index was calculated for the PFI-RSI benchmark to determine the cut-off value that optimized the PFI-RSI scale’s ability to discriminate between returning and not returning to the preoperative level of sport when equal weight was given to sensitivity and specificity. Patients who did not participate in sports pre- or postoperatively were excluded from this analysis as well as the return sport analysis.

Spearman’s rank-order correlation was used to assess the correlation between the PFI-RSI scale and outcome parameters (BPII 2.0, Kujala score, VAS for pain, TAS, subjective satisfaction) and demographic factors (patients’ age at the time of surgery and follow-up time). Statistical significance was set at a *p* value of < 0.05.

To assess the statistical power of this study, a post hoc power analysis was performed for the group difference (return to preoperative level of sport vs. no return to preoperative level of sport) regarding the PFI-RSI scale using two-tailed t tests. It was shown that the included sample size could achieve an adequate power of > 0.999 with an alpha of 0.05 (G*Power 3.1.9.6, Düsseldorf, Germany) [26].

## Results

Follow-up examinations were conducted in 65 patients at a median 61.0 (40.0-78.5) months postoperatively. Of those, 32 (49.2.%) underwent previous surgeries before undergoing DFO at the authors’ institution. Detailed patient demographics are given in Table 1.

**Table 1:** Patient demographics

	Mean ± SD or No. (%)
No. of patients (knees)	65 (73)
Age at time of surgery (years)	23.0^a^ (19.0–29.0)
Sex (female/male)	46/19 (70.8% female)
No. of patients with previous surgeries for PFI	32 (49.2%)
Follow-up (months)	61.0^a^ (40.0-78.5)

Normally distributed continuous variables are shown as mean ± standard deviation. Non-normally distributed continuous variables are shown as median (25–75% interquartile range). *PFI* Patellofemoral instability. ^a^Values are median

### Surgical procedures

In 27 cases (41.5%), the DFO aimed to correct an increased femoral antetorsion and in 22 cases (33.8%), the DFO aimed to correct valgus malalignment. In the remaining cases (16; 24.6%) the DFO aimed to correct both valgus and torsional malalignment. Concomitant procedures were performed in 54 cases (83.1%). Most commonly, the DFO was combined with MPFL reconstruction (43 cases; 66.2%), trochleoplasty (8 cases; 12.3%) and/or tibial tubercle osteotomy (4 cases; 6.2%). By follow-up, 47 patients (72.3%) underwent implant removal.

### Return to sport

Of the patients that participated in sports preoperatively, 24 patients (40.7%) returned to their preoperative level of sport and 35 patients (59.3%) did not reach their preoperative level of sport again. Reasons for an inability to return to the preoperative level of sport are presented in Table 2. Nonetheless, the majority of patients reported that their overall sporting ability either improved (29 patients; 44.6%) or did not change (14 patients; 21.5%) following surgery.

**Table 2:** Reasons for an inability to return to the preoperative level of sports

	No. (%)
Fear of re-injury	24 (68.6%)
Pain	24 (68.6%)
Instability persistence	22 (62.9%)
Lack of confidence in the knee joint	22 (62.9%)
Other injury-dependent factors	8 (22.9)
Other	1 (2.9%)

### Clinical and functional outcome

**Fig. 1:**
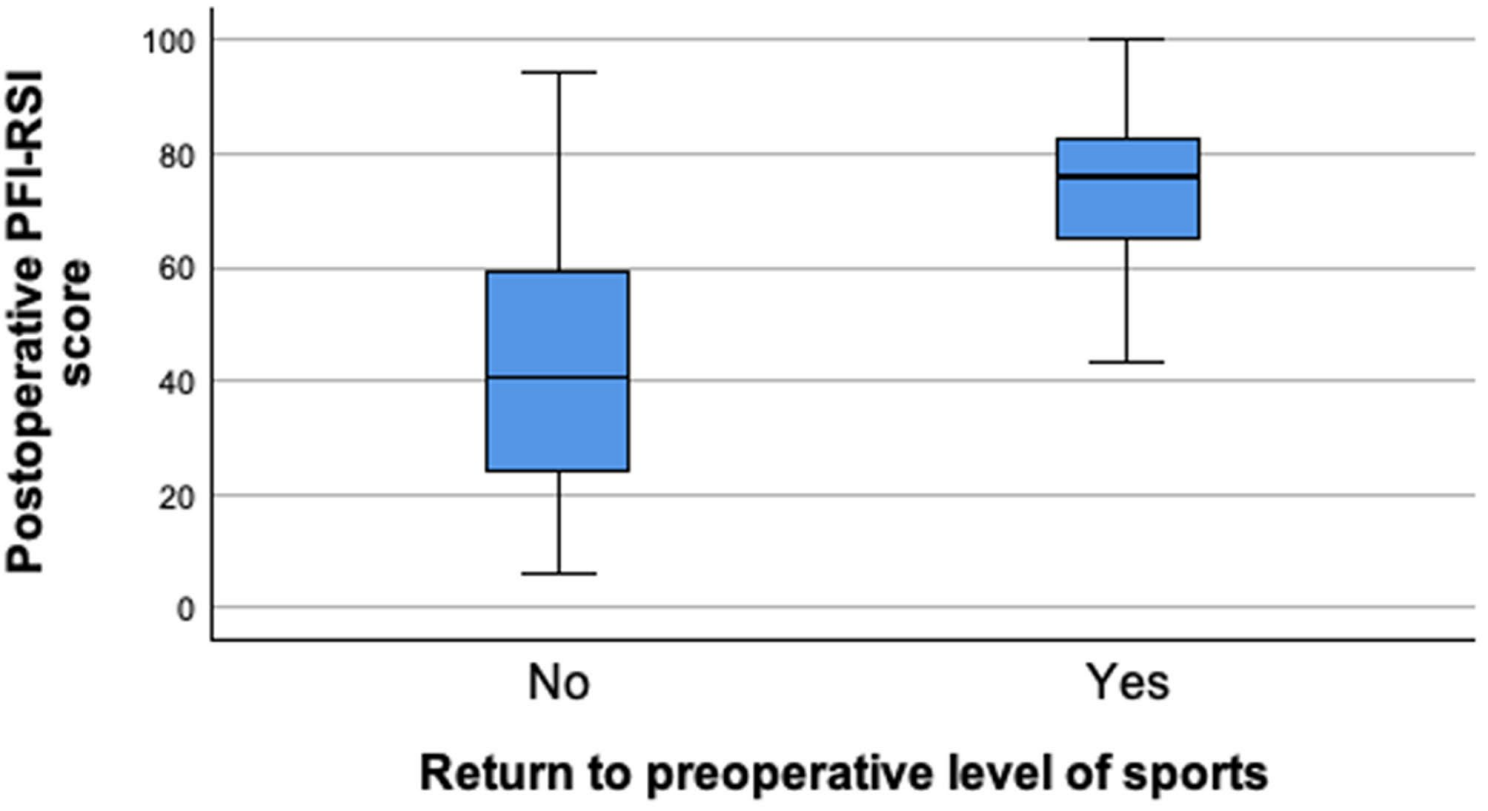
Box plot showing the PFI-RSI score in patients who did return to the preoperative level of sports (left) vs. patients who did not (right). *PFI-RSI* Patellofemoral Instability-Return to Sport after Injury

Functional outcome and satisfaction with the postoperative result (8.0 [5.0-9.8]) were favorable (Table 3). A significant higher PFI-RSI score was observed in patients that returned to their preoperative level of play compared to those that did not (75.8 [64.4–84.2] vs. 40.8 [23.4–60.9], *p* < 0.001; Fig. 1).

**Table 3:** Patient-reported outcome measures at follow-up

	Median (25–75% interquartile range)
Banff Patella Instability Instrument 2.0	66.1 (36.7–84.7)
Kujala score	81.0 (67.0–89.0)
Tegner Activity Scale	4.0 (3.0-4.3)
Visual Analog Scale for pain	1.0 (0–2.0)

**Fig. 2:**
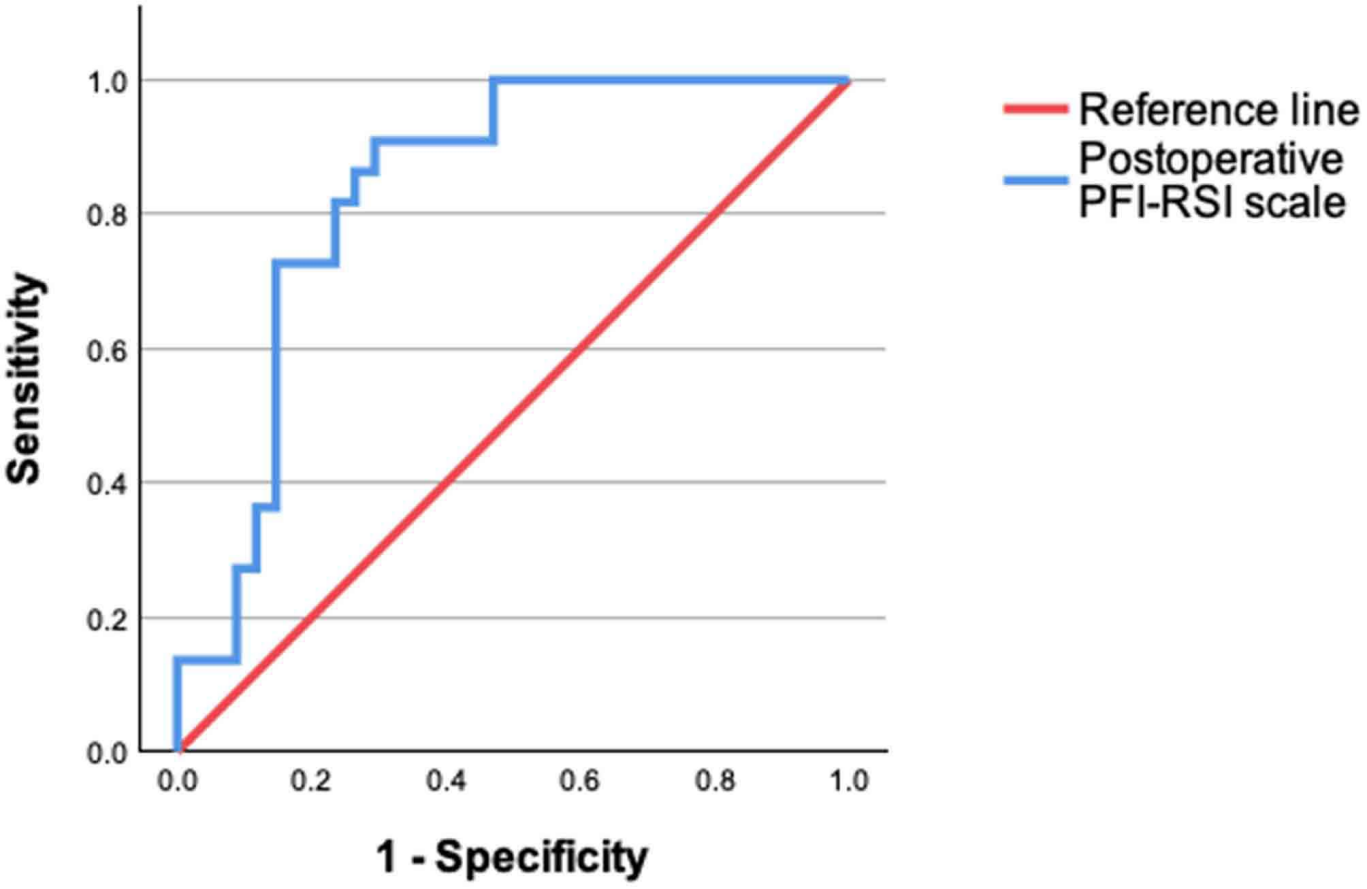
Receiver operating characteristic analysis for the PFI-RSI score and its ability to discriminate between return to preoperative level of levels of sports vs. no return to preoperative level sports. Area under curve = 0.834. *PFI-RSI* Patellofemoral Instability-Return to Sport after Injury

Receiver operating characteristic curve analysis revealed an area under the curve of 0.834 (Fig. 2). A PFI-RSI value of 55 had the highest Youden index value (0.615), which represented a sensitivity of 90.9% and a specificity of 70.6% in discriminating whether return to the preoperative level of sport was achieved or not.

**Fig. 3:**
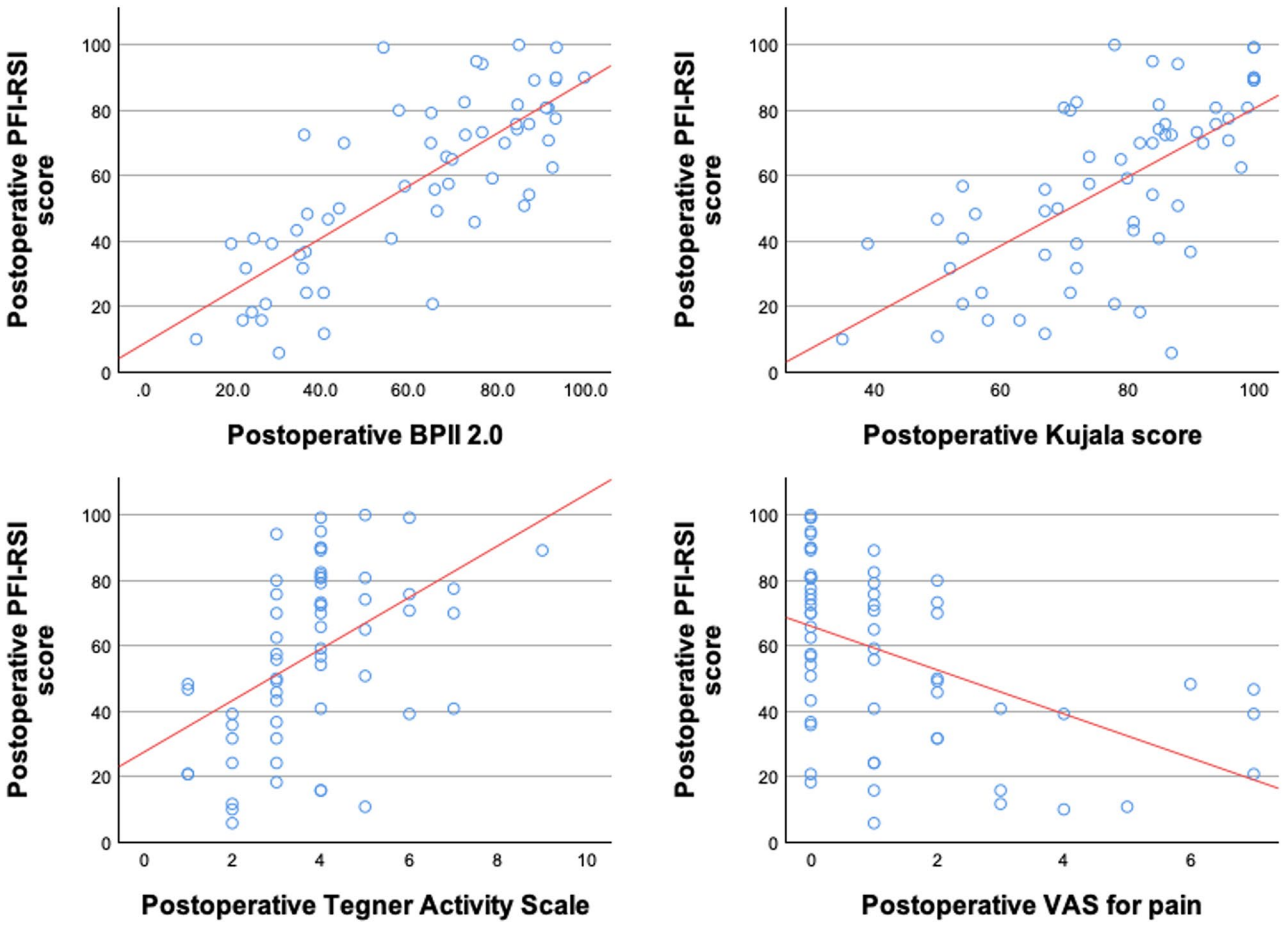
Correlation analyses between the PFI-RSI scale and the BPII 2.0 (upper left), Kujala score (upper right), TAS (bottom left) and VAS for pain (bottom right). A strong correlation was seen between the PFI-RSI scale and BPII 2.0. Moderate correlations were observed between the PFI-RSI scale and the Kujala score, TAS and VAS for pain. *BPII 2.0* Banff Patella Instability Instrument 2.0, *PFI-RSI* Patellofemoral Instability-Return to Sport after Injury, *TAS* Tegner Activity Scale, *VAS* Visual Analog Scale

The PFI-RSI scale showed moderate to strong correlations with PROM (Fig. 3) and weak correlations with patient satisfaction as well as follow-up time. The correlation with follow-up time was, however, not statistically significant (*p* = 0.123). No correlation appeared between the PFI-RSI scale and patients’ age at the time of surgery (Table 4). No significant difference was observed between male and female patients regarding the PFI-RSI scale (*p* = 0.187). Postoperative instability recurrence was reported in 8 cases (12.5%). These patients reported a significantly lower PFI-RSI score than those that did not suffer from dislocation recurrence (26.3 [16.4–68.6] vs. 65.0 [42.1–80.4], *p* = 0.042). Ten patients (15.4%) underwent revision surgery by follow-up, three times due to loss of correction and instability recurrence each, two times due to infection and one time due to arthrofibrosis and early implant-related irritation each.

**Table 4:** Correlation analyses between the PFI-RSI scale and PROMs, patient’s satisfaction, follow-up time and patient-related data

	Spearman’s rank-order correlation (ρ)	*p* value
PROM		
BPII 2.0 Kujala score TAS VAS for pain	0.773 0.658 0.511 − 0.523	**< 0.001** **< 0.001** **< 0.001** **< 0.001**
Satisfaction	0.321	**0.012**
Follow-up time	0.200	0.123
Age at the time of surgery	0.066	0.612

*BPII 2.0* Banff Patella Instability Instrument 2.0, *PROM* patient-reported outcome measures, *TAS* Tegner Activity Scale, *VAS* Visual Analog Scale. Bolded *p* values indicate statistical significance

## Discussion

The most important finding of this study was that in patients with high-grade PFI treated by DFO, patients who returned to their preoperative level of sport scored significantly higher on the PFI-RSI scale than patients who did not. Further, the PFI-RSI scale was able to discriminate between patients that returned to the preoperative level of sport and those who did not, indicating that psychological readiness may play a prominent role on the ability to return to sport in this patient cohort. A cut-off value for the PFI-RSI score of 55 was determined to be able to identify whether or not patients return to the preoperative level of sport with an excellent sensitivity and good specificity. The PFI-RSI scale showed strong correlations with postoperative knee function and knee-related quality of life and moderate correlations with sporting activity as well as pain levels. Similar to the rehabilitation following other knee injuries/surgeries, such as ACL reconstruction [27], it seems imperative to screen for maladaptive psychological responses in this cohort. This screening identifies patients at risk for not returning to the preoperative level of sport as PROM alone provide only little information about return to sports rates and patients’ readiness to return to sport [28].

The applicability of the Return to Sport after Injury scale in patients with PFI has previously been investigated in a patient cohort who underwent MPFL reconstruction. In their retrospective analysis, Hurley et al. reported that the majority of patients who did not return to their preoperative level of play reported low MPFL-RSI scores [6]. However, as only patients that did not return to play were included in their analysis, extrapolation of their data may be limited. In the current study, both patients that did as well as patients that did not return to the preoperative level of sport were included, which made the calculation of a cut-off value for the PFI-RSI scale to predict return to play feasible.

Interestingly, the cut-off value for the RSI scale with the highest Youden index in the current study is highly comparable to the cut-off value of 56 points reported in patients after ACL reconstruction [27]. This suggests that a similar degree of psychological readiness is needed for both patient groups to return to the pre-injury/preoperative level of sport. This similarity may in part be related to the fact that recurrent PFI affects knee function as much as ACL deficiency does [29]. Further, both in the present study as well as in patients that underwent ACL reconstruction, the RSI scale correlated with knee function and pain levels [30, 31]. Correlations between the adapted RSI scale and PROM were also found in other pathologies across different joints [19–21, 32].

While only 40.7% of patients returned to their preoperative level of sport, the majority of patients reported either an improvement or no change in sporting ability. This discrepancy between return to sport and patient-reported athletic ability was also observed in other studies evaluating return to sports after surgery for PFI. It may occur in part due to sports and/or lifestyle modifications [4, 33]. Mengis et al. [33] reported that 42% of patients undergoing trochleoplasty returned to the preoperative level of sport with a higher likelihood reported for patients with preoperative lower preoperative activity levels (TAS ≤ 4). They reported lifestyle changes – potentially sporting activities becoming less important to them – to confound the low rate of return to the previous level of sport. The latter was also reported in a systematic review assessing return to sport following MPFL reconstruction where 27.4% of patients with a lower postoperative level of play reported a loss of interest or lack of time [4]. Overall, however, fear of re-injury (along with pain) was the most common reason in the present study for patients not being able to return their preoperative level of sport. This outcome is consistent with the findings of previous studies assessing return to sport in patients that underwent surgery for PFI [4, 6, 33].

The current study has several limitations. First, because of its retrospective nature, a possible selection bias cannot be ruled out. Second, the study included both patients with primary DFO as well as DFO as a revision procedure after previously failed surgery, derotational and/or varus correcting DFO as well as additional procedures and different types and levels of sport, thus, introducing a possible heterogeneity of the study population. This may, however, be related to the nature of PFI being a complex and heterogeneous entity. Third, the PFI-RSI scale is not fully validated according to Consensus-based Standards for the selection of health status Measurement Instruments (COSMIN). In this context, no testing for internal consistency or retest-reliability was performed, which is comparable to Hurley et al. [6]. Nonetheless, the PFI-RSI scale is a close adaptation of the validated and well-established ACL-RSI scale. Lastly, all outcomes measures were only evaluated postoperatively. Consequently, this study cannot comment on the value of assessing the PFI-RSI scale preoperatively as to predict postoperative return to sport. This may be of interest for future studies.

The present findings indicate that the PFI-RSI scale could serve as a valuable tool for the psychological assessment of patients undergoing DFO for the treatment of complex PFI who aim to return to sport postoperatively and to identify those at risk.

## Conclusion

Psychological readiness to resume sporting activities following DFO for the treatment of PFI was significantly higher in patients who returned to their preoperative level of play than in patients who did not. Psychological readiness correlated with functional outcome. Postoperative rehabilitation should focus both on physical and psychological factors. In patients with low PFI-RSI scores following the first postoperative year, additional psychological support may be beneficial.

## Data Availability

The data that support the findings of this study are available on request from the corresponding author. The data are not publicly available due to privacy or ethical restrictions.
